# P-1125. Surgical Site Infection Surveillance in Tennessee, 2015 – 2023

**DOI:** 10.1093/ofid/ofaf695.1320

**Published:** 2026-01-11

**Authors:** Ashley Gambrell, Marissa M Turner, Jordan Morris, Vicky Lindsey, Christopher Wilson

**Affiliations:** Tennessee Department of Health, Nashville, TN; Tennessee Department of Health, Nashville, TN; Tennessee Department of Health, Nashville, TN; Tennessee Department of Health, Nashville, TN; Tennessee Department of Health, Nashville, TN

## Abstract

**Background:**

Nationally, surgical site infections (SSIs) cause significant morbidity and mortality. SSIs lengthen hospital stays, increase readmissions, and cost from $3.5 to $10 billion yearly. Understanding SSI occurrence allow for better infection prevention and understanding of associated risk factors. Tennessee (TN) acute care hospitals are required to report all SSIs assigned to abdominal hysterectomies (HYST), colon surgeries (COLO), and coronary artery bypass grafts (CABG) by National Healthcare Safety Network (NHSN) surveillance protocols. We utilized these data to look at SSIs across TN and evaluate characteristics of patients and procedures.

**Methods:**

Data from NHSN included adult cases, 18 years or older, from 2015 – 2023 associated with NHSN-defined CABGs, HYSTs, and COLO surgeries. Patient and procedure characteristics collected from NHSN SSI forms were assessed using Chi-Square Independence tests and one-way ANOVAs, using SAS v9.4.

**Results:**

There were 5,652 SSIs total, shown in Table 1. Most cases did not have diabetes (68.51%), did not have secondary bloodstream infections (96.53%), and did not die during admission (96.60%). Most SSIs, 3,613 (63.92%) cases, were attributed to COLO surgeries. Most SSIs, 2,546 (45.05%) cases, were detected in patients upon readmission to the facility where the procedure was performed. 807 (14.28%) cases had documented infections in the surgical space at the time of surgery. Despite hospital access to all patient demographics, most patients were recorded as “unknown” race (91.42%). All analyzed patient characteristics varied significantly across procedure type, as shown in Tables 1 – 3, except the number of SSIs contributing to a patient’s death.

**Conclusion:**

Except contribution of SSI to death, all characteristics available for analysis from NHSN were significantly different across observed procedure types for TN SSIs. While NHSN factors in demographic data known to contribute to SSIs for assessing patient risk, there were additional significant differences in infection acquisition based on the procedure type. Analysis was impeded on some patient characteristics due to a lack of data such as race and ethnicity. Better documentation and more robust reporting practices would assist in understanding significant risk factors for SSIs.

**Disclosures:**

All Authors: No reported disclosures

